# *Moraxella catarrhalis* Macrolide-Resistant Isolates Are Highly Concentrated in Two MLST Clonal Complexes -CCN10 and CC363

**DOI:** 10.3389/fmicb.2017.00201

**Published:** 2017-02-10

**Authors:** Ya-Li Liu, Meng Xiao, Jing-Wei Cheng, He-Ping Xu, Zhi-Peng Xu, Sha Ye, Wen-Juan Zhang, Timothy Kudinha, Fanrong Kong, Ying-Chun Xu

**Affiliations:** ^1^Department of Clinical Laboratory, Peking Union Medical College Hospital, Peking Union Medical College, Chinese Academy of Medical SciencesBeijing, China; ^2^Department of Clinical Laboratory, The First Affiliated Hospital of Xiamen UniversityXiamen, China; ^3^Department of Clinical Laboratory, Bazhou People’s HospitalXinjiang, China; ^4^Department of Clinical Laboratory, Beijing Youan Hospital, Capital Medical University/Hospital for Infectious Diseases of BaodingHebei, China; ^5^Charles Sturt University, Orange Campus, OrangeNSW, Australia; ^6^Centre for Infectious Diseases and Microbiology Laboratory Services, ICPMR – Pathology West, Westmead Hospital, University of Sydney, WestmeadNSW, Australia

**Keywords:** *Moraxella catarrhalis*, *copB*, mlst, clonal complexes, macrolide-resistance

## Abstract

To gain some insights into the molecular evolution of *Moraxella catarrhalis* macrolide resistance, PCR and sequencing analysis of the 23S rRNA gene, *copB* typing and multilocus sequence typing (MLST) were performed on 181 *M. catarrhalis* isolates. The isolates were obtained from children (*n* = 47) and adults (*n* = 134) presenting with respiratory disease in the years 2010–2014. Macrolide resistance was highly age-related, and nucleotide position alterations at A2330T could be detected in all macrolide-resistant isolates. *copB* 0 and *copB* NT (non-typable) were only found in macrolide-susceptible isolates from adults. Furthermore, *copB* I/III was the main type in adult or macrolide-susceptible isolates, while *copB* II was the most common type in children or macrolide-resistant isolates. Twenty-two different MLST clusters (sharing 7 of the 8 identical loci) were detected and only four likely primary founders (ST224, ST363, STN08, and STN10) which belong to clonal complex (CC) 224, CC363, CCN08, and CCN10, were detected, respectively. Macrolide-resistant *M. catarrhalis* isolates were highly concentrated in two CCs (CCN10 and CC363), which indicates some potential evolutionary advantage or co-evolution to some extent. However, further studies are needed to fully elucidate the evolution of CCN10 and CC363 in macrolide resistance.

## Introduction

*Moraxella catarrhalis* is a prominent pathogen that causes acute otitis media in children and lower respiratory tract infections in adults (such as exacerbations of chronic obstructive pulmonary disease) (Hol et al., 1996; Ahmad, 1998), resulting in significant socio-economic burden on healthcare systems globally.

Previous studies, including our own (Wang et al., 2011; Flamm et al., 2012; Liu et al., 2012, 2015; Tang et al., 2016), have reported on the increased prevalence of macrolide-resistant *M. catarrhalis* in Mainland China, ranging from 40 to 80% in children, and 5–10% in adults. A better understanding of the evolutionary path of the organism from macrolide-susceptible to macrolide-resistant *M. catarrhalis*, is crucial for controlling the spread of macrolide resistance. However, based on our previous studies, showing 40–60% macrolide resistance amongst *M. catarrhalis* isolates from healthy toddlers (12–18 months) (Liu et al., 2012), and that most macrolide resistant isolates (5/7) were obtained from patients who had not received macrolide antibiotics within the previous 30 days (Liu et al., 2015), we think that macrolide resistance is acquired.

Multilocus sequence typing (MLST) has been widely employed in epidemiological investigations of various scales (Cox et al., 1995; Enright and Spratt, 1998; Maiden et al., 1998; Enright et al., 2001; Qin et al., 2009), including population, pathogenicity, and evolution studies, of several bacteria. To gain insights into the molecular evolution of macrolide resistance in *M. catarrhalis*, PCR and sequence analysis of the 23S rRNA gene, *copB* typing, MLST, and macrolide susceptibility testing, were performed on 181 *M. catarrhalis* isolates.

## Materials and Methods

### Statement

All the authors confirm that all experiments were performed in accordance with relevant guidelines and regulations. Only clinical bacterial isolates, and no human subjects (including the use of tissue samples), were used in the present study. Thus informed consent was not obtained from the patients. The study was approved by the Human Research Ethics Committee of Peking Union Medical College Hospital (No. S-424).

#### Bacterial Isolates

One hundred and twenty non-duplicate *M. catarrhalis* isolates were collected from the sputum or broncho-alveolar lavage of adult patients (>18 years of age) in the Peking Union Medical College hospital (PUMCH) (59 wards) between 2010 and 2013. Furthermore, 14 *M. catarrhalis* isolates from the sputum or ear purulent secretion of adult patients (10 wards), and 47 from the sputum or ear purulent secretion of children (7 wards), were obtained from the First Affiliated Hospital of Xiamen University (FAHXU) in 2014. The isolates were identified by mass spectrometry (MALDI-TOF MS) Bruker Biotyper (Bruker Daltonics, Bremen, Germany). Isolates were stored at -70°C until testing, with a minimum number of passages.

#### Macrolide Susceptibility Testing

The isolates were tested for susceptibility to erythromycin and azithromycin using the Kirby-Bauer disk diffusion (Thermo Fisher, Oxoid) method in accordance with the CLSI M45-A3 guideline (Clinical and Laboratory Standards Institute [CLSI], 2015). In addition, the E-test method was used to confirm the antimicrobial susceptibility results of macrolide-resistant *M. catarrhalis*. *Staphylococcus aureus* ATCC 25923 and ATCC 29213 were used for quality control.

#### *copB* Polymerase Chain Reaction-Restriction Fragment Length Polymorphisms

The *copB* gene was tested for in all the isolates (*n* = 181), as described by Verhaegh et al. (2008) and Liu et al. (2012). A touchdown thermocycling program was used for all PCRs. The touchdown protocol used an initial annealing temperature of 70°C, which was reduced by 1°C per cycle over 15 cycles of PCR. The final 20 amplification cycles used an annealing temperature of 55°C. The *copB* PCR products were digested with *RsaI* (New England Biolabs, Ipswich, MA, USA). Five units of *RsaI* were used per reaction mix, and the mixture was incubated at 37°C. Agarose gels were prepared at a 1% concentration in 0.5 × TBE buffer (45 mM Tris base, 45 mM boric acid, and 1 mM EDTA). DNA fragments were separated using an electric current of 120 V/cm RT for 30 min. Gels were stained for 15–20 min in ethidium bromide (1 mg/ml) and decolorized in distilled water for 2–5 min. Fluorescent bands were visualized using UV transillumination, and gel images were captured using Bio-Rad GelDocEQ (Bio-Rad). A 50 bp Ladder (NEB) was used as a molecular size standard (TransGen Biotech, Beijing, China). Four types of *copB* were included: type 0, consisting of 374 and 157-bp; *copB* types I/III, consisting of 342 and 157-bp; *copB* type II, with bands of 332 and 187-bp; and *copB* type IV, consisting of a single band of 519-bp.

#### PCR and Sequencing Analysis of the 23S *rRNA* Gene

The 23S *rRNA* gene was amplified and sequenced using primer pairs described before (MUT-F, 5′-^2685^CAGGCTGCTGCAACTGTTTA^2704^-3′, and MUT-R, 5′-^3618^CAACCGAAACA CCAGAGGTT^3599^-3′, from *M. catarrhalis* KCCM: 40056; positions determined according to the bases on GenBank submission of FJ410380) (Liu et al., 2012, 2016).

#### Multilocus Sequence Typing

Multilocus sequence typing was performed on all the 181 isolates by amplification of internal fragments of the housekeeping genes *glyRS* (glycyl-tRNA synthetase beta subunit), *ppa* (Pyrophosphate phospho-hydrolase), *efp* (elongation factor P), *fumC* (fumarate hydratase), *trpE* (anthranilate synthase component I), *mutY* (adenine glycosylase), *adk* (Adenylate kinase), and *abcZ* (ATP-binding protein), in separate PCRs. Based on the sequences, alleles were determined by querying the central database at http://mlst.warwick.ac.uk/mlst/dbs/Mcatarrhalis/ Finally, a graphical representation of the relatedness of isolates was performed using the program enhanced based upon BioNumerics (version 7.6). Through the availability of an MLST plugin, the BioNumerics program automatically analyses batches of sequence trace files, connects to online MLST databases, retrieves corresponding allele numbers, sequence types, as well as available clonal complex (CC) information.

### Statistical Analysis

The prevalence of macrolide-resistant *M. catarrhalis* in adults and children, the frequency of sequence types (STs) or the *copB* types in adults and children, or the frequency of STs or the *copB* types in macrolide-susceptible and resistant isolates, was statistically compared using Pearson’s χ^2^-test (or the Fisher exact test, when appropriate) for categorical data.

## Results

### *M. catarrhalis* Genetic Population Study

**FIGURE 1:**
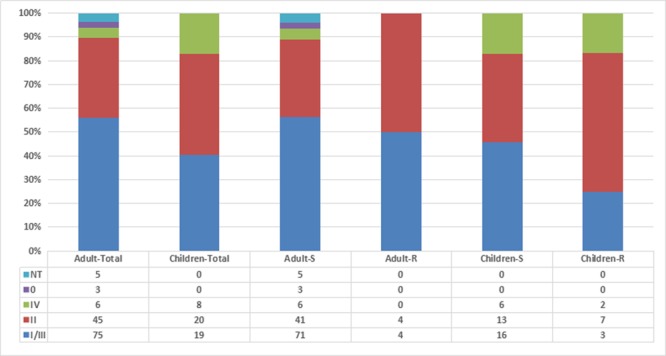
**Relationship among *Moraxella catarrhalis* isolates by age, antibiotic resistance, and *copB* typing.** Adult-S: macrolide-susceptible *M. catarrhalis* isolates from adults, Adult-R: macrolide-resistant *M. catarrhalis* isolates from adults, Children-S: macrolide-susceptible *M. catarrhalis* isolates from children, Children-R: macrolide-resistant *M. catarrhalis* isolates from children.

Among the 181 *M. catarrhalis* isolates studied, 20 were resistant to macrolide (MIC > 256 μg/ml). Furthermore, a nucleotide position alteration at A2330T was detected in each of the 20 macrolide-resistant isolates. The prevalence of macrolide-resistant *M. catarrhalis* in adults was much lower (6.0%, 8/134) than in children (25.5%, 12/47; *p* = 0.006). Using the *copB* PCR-RFLP typing method, *copB* II was the most common type amongst the resistant isolates (11/20, 55%), whilst only 33.5% *copB* II (54/161) was detected in macrolide-susceptible isolates (*p* = 0.082) (**Figure 1**). *copB* I/III (56.0%, 75/134) was the main type in isolates from adults while only 40.4% *copB* I/III (19/47) was detected in isolates from children (*p* = 0.089). The *copB* 0 and non-typeable (NT) isolates were only detected in adults (*p* = 0.114).

### Relationship between Age, Macrolide Resistance, and MLST Results

Using MLST, the 181 isolates were discriminated into 119 different ST, including 103 novel STs (ST293-ST327, ST329-369, STN01-STN31) (STN01-STN31 were defined in the present study). In adults, ST224, ST105, and ST180 (4.5%, 6/134 each), were the most common STs, followed by STN05 and STN26 (3.7%, 5/134, for each), STN29 and ST308 (3.0%, 4/134, for each), ST64, STN14, ST62, ST156, and ST176 (2.2%, 3/134, for each). Just like in adults, ST224 (8.5%, 4/47) was the most common ST in children, albeit almost doubled in prevalence, followed by ST64 and ST342 (4.3%, 2/47, for each). In PUMCH, only isolates from adults were included in the study whilst in FAHXU, children were the main population. However, even when the isolates from adults and children in FAHXU were divided into two parts, the frequency of STs was almost the same as mentioned above.

**FIGURE 2:**
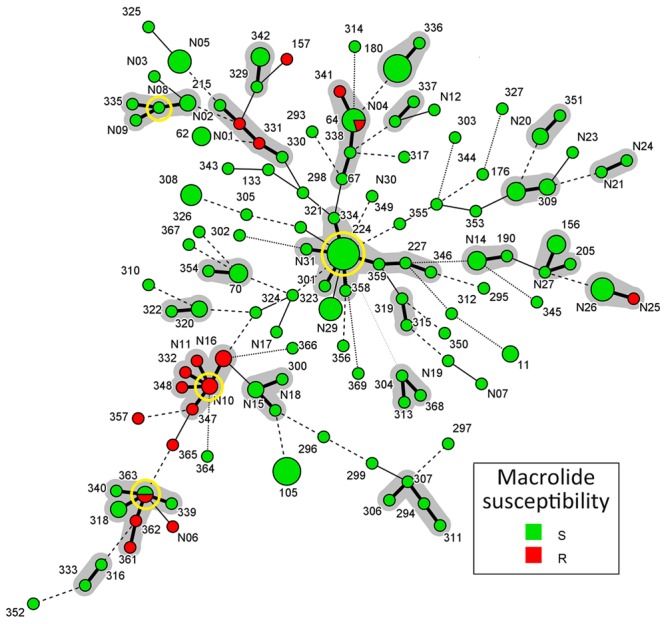
**Population snapshot of *M. catarrhalis* based on allelic profiles of MLST.** Sequence types (STs) that shared alleles at ≥5 of the eight MLST loci were obtained from the *M. catarrhalis* MLST website, and a diagram was constructed by using Bionumerics. Each circle in Figure corresponds to a MLST ST, and different circle colors represent different susceptibilities to macrolide. The lines between circles indicate the similarity between profiles: bold line, seven of eight MLST alleles/MLVA loci in common; normal line, six alleles/loci in common; dashed line, five alleles/loci in common; dotted line, ≤4 alleles/loci. The gray halo surrounding the STs in Figure denotes STs belonging to different MLST clusters. The likely primary founders with at least three links to other STs are positioned centrally in the cluster and identified by yellow rings. Cluster 1: ST363/ST340/ST339/ST362/ST361/ST318, Cluster 2: STN10/STN11/STN16/ST332/ST348/ST347, Cluster 3: STN08/ST335/ST215/STN09, Cluster 4: ST224/ST227/ST323/ST334/ST346/ST358/ ST359/STN31, Cluster 5: STN25/STN26, Cluster 6: ST330/ST331/STN01/STN02, Cluster 7: ST64/ST67/ST338/ST341, Cluster 8: ST329/ST342, Cluster 9: ST180/ST336, Cluster 10: ST337/STN04, Cluster 11: STN20/ST351, Cluster 12: STN21/STN24, Cluster 13: ST176/ ST309, Cluster 14: ST156/ST205/STN27, Cluster 15: ST190/STN14, Cluster 16: ST315/ST319, Cluster 17: ST304/ST313/ST368, Cluster 18: ST294/ST306/ST307/ST311, Cluster 19: ST337/STN04, Cluster 20: ST300/STN15/STN18, Cluster 21: ST320/ST322, Cluster 22: ST70/ST354.

A population snapshot of the studied *M. catarrhalis* isolates based on allelic profiles of MLST is shown in **Figure 2**. STs that shared alleles at ≥5 of the 8 MLST loci were obtained from the *M. catarrhalis* MLST website, and a diagram was constructed using Bionumerics. There are 22 different MLST clusters (Definition: Sharing 7 of the 8 identical loci, or single locus variant with one other member of the group) marked in **Figure 2** using gray halo surrounding the MLST STs. But only four likely primary founders (Definition: A ST which is positioned centrally in the cluster and with at least three links to other STs) (ST224, ST363, STN08, and STN10) were identified and are marked by yellow rings. The four likely primary founders (ST224, ST363, STN08, and STN10) were defined to belong to CC (Definition: A cluster containing primary founder) CC224, CC363, CCN08, and CCN10. In the 22 different MLST clusters, there were only five clusters that contained macrolide-resistant *M. catarrhalis* isolates in which only CCN10 and CC363 could be likely defined for diverse STs and few links. The CCN10 contained six STs (STN10, STN11, STN16, ST332, ST347, and ST348) and all the STs consisted of macrolide-resistant isolates. The CC363 contained six STs (ST363, ST318, ST339, ST340, ST361, and ST362) and the likely primary founder ST363 consisted of both macrolide-resistant and susceptible isolates.

## Discussion

CopB is one of the several *M. catarrhalis* outer membrane proteins that elicit a systemic humoral immune response in humans, resulting in the production of serum antibody specific for CopB, which underscores the potential of CopB as a vaccine candidate (Meier et al., 2003; Murphy et al., 2005; Liu et al., 2006). The *copB* gene is relatively conserved in *M. catarrhalis*, and has been detected in all isolates examined so far (Liu et al., 2012, 2015). Digestion of the *copB* products with *RsaI* restriction enzyme divides the organism into four *copB* types, namely *copB* type 0 (374 and 157 bp), type I/III (342 and157 bp), type II (332 and 187 bp) and type IV (519 bp) (Verhaegh et al., 2008), and has already been used in several typing studies (Verhaegh et al., 2008; Liu et al., 2012, 2015).

In our previous studies, we found that there was a trend toward an increase in *copB* types I/III, and a decrease in *copB* type II, in adult associated *M. catarrhalis* isolates, when compared with isolates from children (Liu et al., 2012, 2015). The present findings are somewhat in agreement to these previous results although the difference was not statistically significant, which suggests that the *copB* type may not be age-related.

When the allelic profiles of MLST were analyzed using Bionumerics software, population diversity was clearly demonstrated (**Figure 2**). However, four likely primary founders (ST224, ST363, STN08, and STN10) were demonstrated in CCs CC224, CC363, CCN08, CCN10. The predicted primary founder ST224, with the largest number and complicated links to other STs, is likely to be the ancestor of all macrolide- susceptible isolates. And all the five clusters encompassing macrolide-resistant *M. catarrhalis* isolates, located at the edge of the population snapshot (**Figure 2**) with less links to CC224, seem to have evolved in several ways from macrolide-susceptible isolates, but not exactly only from CC224.

One important finding of this study is the description of two macrolide-resistant CCs, CCN10 and CC363, and the likely primary founders STN10 and ST363. As illustrated in **Figure 2**, the two valuable macrolide-resistant CCs, CCN10 and CC363, are significantly separated from other clusters including CC224, and from the year collected. STN10 and ST224 were first isolated in 2010 and 2012, respectively, from which we surmise that CCN10 is less likely to have evolved from CC224. The small number of isolates analyzed over a short duration (2010–2014) makes it difficult to come up with firm conclusions. Some valuable insights might be gained from studying a large number of isolates collected over a 10 or 12 years period.

A limitation of this study is that the isolates originated from two different hospitals; the prevalence and distribution of macrolide-resistance or STs might be totally different in different areas. Actually, antimicrobial susceptibility testing and MLST typing were first performed on isolates from PUMCH adults, and seven macrolide-resistant isolates and some important STs such as ST224, STN10, and STN16, were detected. This encouraged us to examine whether the macrolide-resistant population was concentrated in selected STs. PUMCH is located in the northern part of China and the patients are mainly adults, while FAHXU is situated in the southern part, and is well known for pediatric infectious diseases. So the two hospitals were ideal locations for our study purpose, including comparing the distribution of different STs and macrolide-resistant isolates in the two hospitals (**Figure 2**; **Supplementary Figure S1**). From the results we can see that even though the distribution of different STs is definitely related to regions, some important STs such as ST224, STN10, and STN16, were detected in both hospitals. Moreover, most STs of macrolide-resistant isolates (except for STN10 and STN16) detected in PUMCH or FAHXU belonged to the same clusters (such as both STN01 and ST331 belonged to cluster 6) (**Figure 2**; **Supplementary Figure S1**), which suggests that these STs might be very critical clones in the process of evolution, especially on macrolide resistance. And we can also see that macrolide resistance is mainly age-related without obvious connection with different regions (**Figure 2**; **Supplementary Figures S1** and **S2**), albeit limited data from the two hospitals. However, even when the results from adults and children in the same hospital were analyzed (**Supplementary Figure S3**), most of the derived conclusions are somehow confirmed.

Further study limitations include the relatively small number of macrolide-resistant *M. catarrhalis* isolates in adults compared with macrolide- susceptible isolates, which may influence the reliability of results to some extent. In addition, STN01–STN31 have not been submitted to http://mlst.warwick.ac.uk/mlst/dbs/Mcatarrhalis/, so these ST names were defined in the present study. Moreover, we compared isolates collected over different time periods in the two hospitals; strains from PUMCH were collected from 2010 to 2013 while the ones from FAHXU were collected in 2014. In our further study, the genomic sequences of these strains would be determined according to the requirements of the Enterobase^1^ instead of the MLST website, and then the accurate STs would be given.

## Author Contributions

Y-LL, MX, TK, and FK wrote the manuscript. J-WC, Z-PX, and H-PX collaborated in molecular investigations of the strains. SY and W-JZ summarized the patient’s medical records. Y-CX designed and supervised the study.

## Conflict of Interest Statement

The authors declare that the research was conducted in the absence of any commercial or financial relationships that could be construed as a potential conflict of interest.

## Footnotes

1 http://enterobase.warwick.ac.uk/species/index/mcatarrhalis

## Supplementary Material

FIGURE S1 **Population snapshot of *M. catarrhalis* based on allelic profiles of multilocus sequence typing (MLST) in two hospitals.** Sequence types (STs) that shared alleles at ≥5 of the eight MLST loci were obtained from the *M. catarrhalis* MLST website, and a diagram was constructed by using Bionumerics. Each circle corresponds to a MLST ST, and different circle colors represent different hospitals. The lines between circles indicate the similarity between profiles: bold line, seven of eight MLST alleles/MLVA loci in common; normal line, six alleles/loci in common; dashed line, five alleles/loci in common; dotted line, ≤4 alleles/loci. The gray halo surrounding the STs denotes STs belonging to different MLST clusters. Cluster 1: ST363/ST340/ST339/ST362/ST361/ST318, Cluster 2: STN10/STN11/STN16/ST332/ST348/ST347, Cluster 3: STN08/ST335/ST215/STN09, Cluster 4: ST224/ST227/ST323/ST334/ST346/ST358/ ST359/STN31, Cluster 5: STN25/STN26, Cluster 6: ST330/ST331/STN01/STN02, Cluster 7: ST64/ST67/ST338/ST341, Cluster 8: ST329/ST342, Cluster 9: ST180/ST336, Cluster 10: ST337/STN04, Cluster 11: STN20/ST351, Cluster 12: STN21/STN24, Cluster 13: ST176/ ST309, Cluster 14: ST156/ST205/STN27, Cluster 15: ST190/STN14, Cluster 16: ST315/ST319, Cluster 17: ST304/ST313/ST368, Cluster 18: ST294/ST306/ST307/ST311, Cluster 19: ST337/STN04, Cluster 20: ST300/STN15/STN18, Cluster 21: ST320/ST322, Cluster 22: ST70/ST354.

FIGURE S2 **Population snapshot of *M. catarrhalis* based on allelic profiles of MLST in adults and children.** Sequence types (STs) that shared alleles at ≥5 of the eight MLST loci were obtained from the *M. catarrhalis* MLST website, and a diagram was constructed by using Bionumerics. Each circle corresponds to a MLST ST, and different circle colors represent different population (A: Adult/C: children). The lines between circles indicate the similarity between profiles: bold line, seven of eight MLST alleles/MLVA loci in common; normal line, six alleles/loci in common; dashed line, five alleles/loci in common; dotted line, ≤4 alleles/loci. The gray halo surrounding the STs denotes STs belonging to different MLST clusters. Cluster 1: ST363/ST340/ST339/ST362/ST361/ST318, Cluster 2: STN10/STN11/STN16/ST332/ST348/ST347, Cluster 3: STN08/ST335/ST215/STN09, Cluster 4: ST224/ST227/ST323/ST334/ST346/ST358/ ST359/STN31, Cluster 5: STN25/STN26, Cluster 6: ST330/ST331/STN01/STN02, Cluster 7: ST64/ST67/ST338/ST341, Cluster 8: ST329/ST342, Cluster 9: ST180/ST336, Cluster 10: ST337/STN04, Cluster 11: STN20/ST351, Cluster 12: STN21/STN24, Cluster 13: ST176/ ST309, Cluster 14: ST156/ST205/STN27, Cluster 15: ST190/STN14, Cluster 16: ST315/ST319, Cluster 17: ST304/ST313/ST368, Cluster 18: ST294/ST306/ST307/ST311, Cluster 19: ST337/STN04, Cluster 20: ST300/STN15/STN18, Cluster 21: ST320/ST322, Cluster 22: ST70/ST354.

FIGURE S3 **Population snapshot of *M. catarrhalis* based on allelic profiles of MLST in adults and children of FAHXU.** Sequence types (STs) that shared alleles at ≥5 of the eight MLST loci were obtained from the *M. catarrhalis* MLST website, and a diagram was constructed by using Bionumerics. Each circle corresponds to a MLST ST, and different circle colors represent different population (A: Adult/C: children). The lines between circles indicate the similarity between profiles: bold line, seven of eight MLST alleles/MLVA loci in common; normal line, six alleles/loci in common; dashed line, five alleles/loci in common; dotted line, ≤4 alleles/loci. The gray halo surrounding the STs denotes STs belonging to different MLST clusters.
