# Supplementary figures and images for: Moraxella catarrhalis Macrolide-Resistant Isolates Are Highly Concentrated in Two MLST Clonal Complexes -CCN10 and CC363
Source: Front Microbiol. 2017 Feb 10;8:201. doi: 10.3389/fmicb.2017.00201 (PMC5300973; doi:10.3389/fmicb.2017.00201)

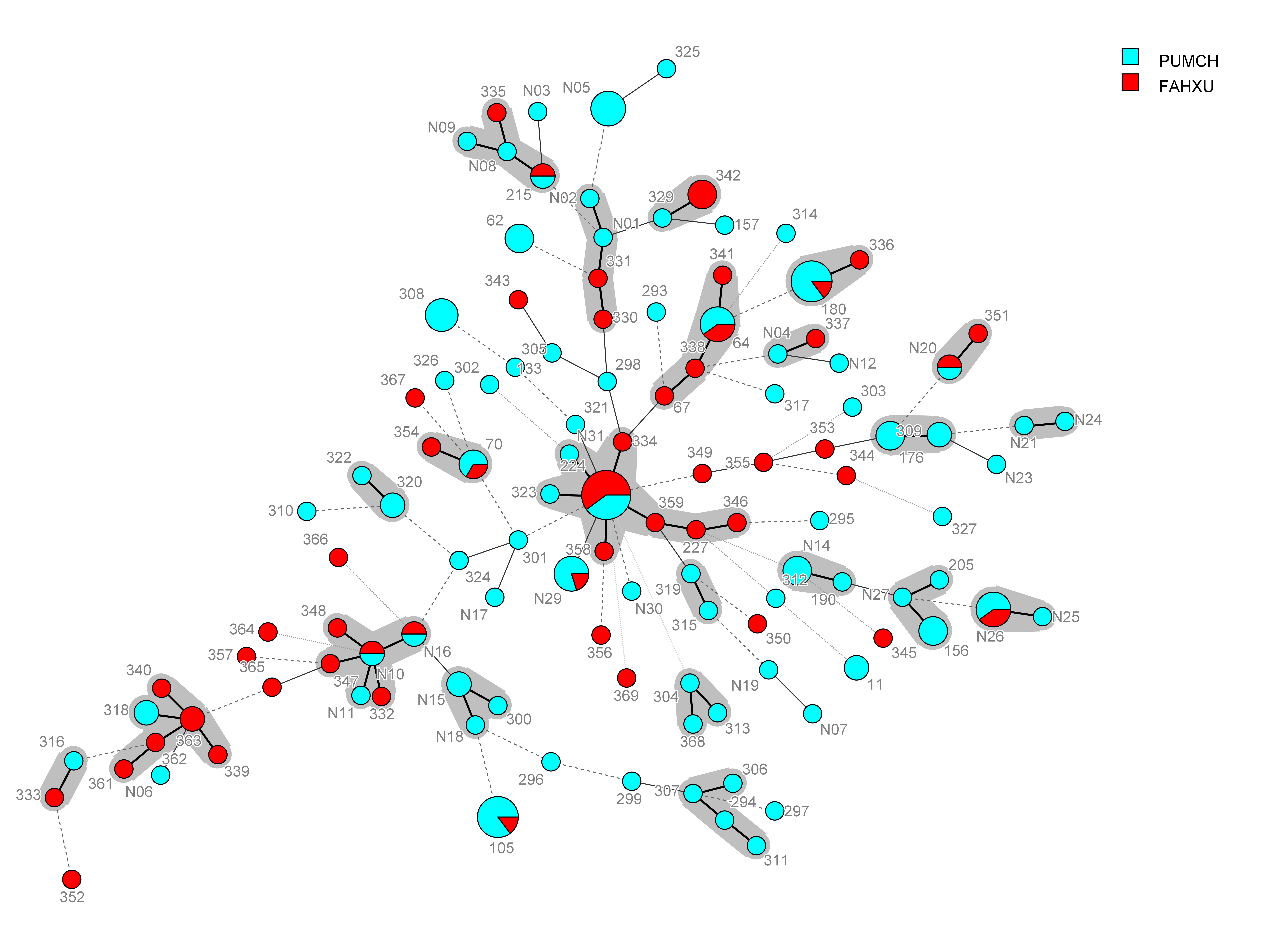

Supplement: FIGURE S1 — Population snapshot of M. catarrhalis based on allelic profiles of multilocus sequence typing (MLST) in two hospitals. Sequence types (STs) that shared alleles at ≥5 of the eight MLST loci were obtained from the M. catarrhalis MLST website, and a diagram was constructed by using Bionumerics. Each circle corresponds to a MLST ST, and different circle colors represent different hospitals. The lines between circles indicate the similarity between profiles: bold line, seven of eight MLST alleles/MLVA loci in common; normal line, six alleles/loci in common; dashed line, five alleles/loci in common; dotted line, ≤4 alleles/loci. The gray halo surrounding the STs denotes STs belonging to different MLST clusters. Cluster 1: ST363/ST340/ST339/ST362/ST361/ST318, Cluster 2: STN10/STN11/STN16/ST332/ST348/ST347, Cluster 3: STN08/ST335/ST215/STN09, Cluster 4: ST224/ST227/ST323/ST334/ST346/ST358/ ST359/STN31, Cluster 5: STN25/STN26, Cluster 6: ST330/ST331/STN01/STN02, Cluster 7: ST64/ST67/ST338/ST341, Cluster 8: ST329/ST342, Cluster 9: ST180/ST336, Cluster 10: ST337/STN04, Cluster 11: STN20/ST351, Cluster 12: STN21/STN24, Cluster 13: ST176/ ST309, Cluster 14: ST156/ST205/STN27, Cluster 15: ST190/STN14, Cluster 16: ST315/ST319, Cluster 17: ST304/ST313/ST368, Cluster 18: ST294/ST306/ST307/ST311, Cluster 19: ST337/STN04, Cluster 20: ST300/STN15/STN18, Cluster 21: ST320/ST322, Cluster 22: ST70/ST354. [file Image_1.TIF]

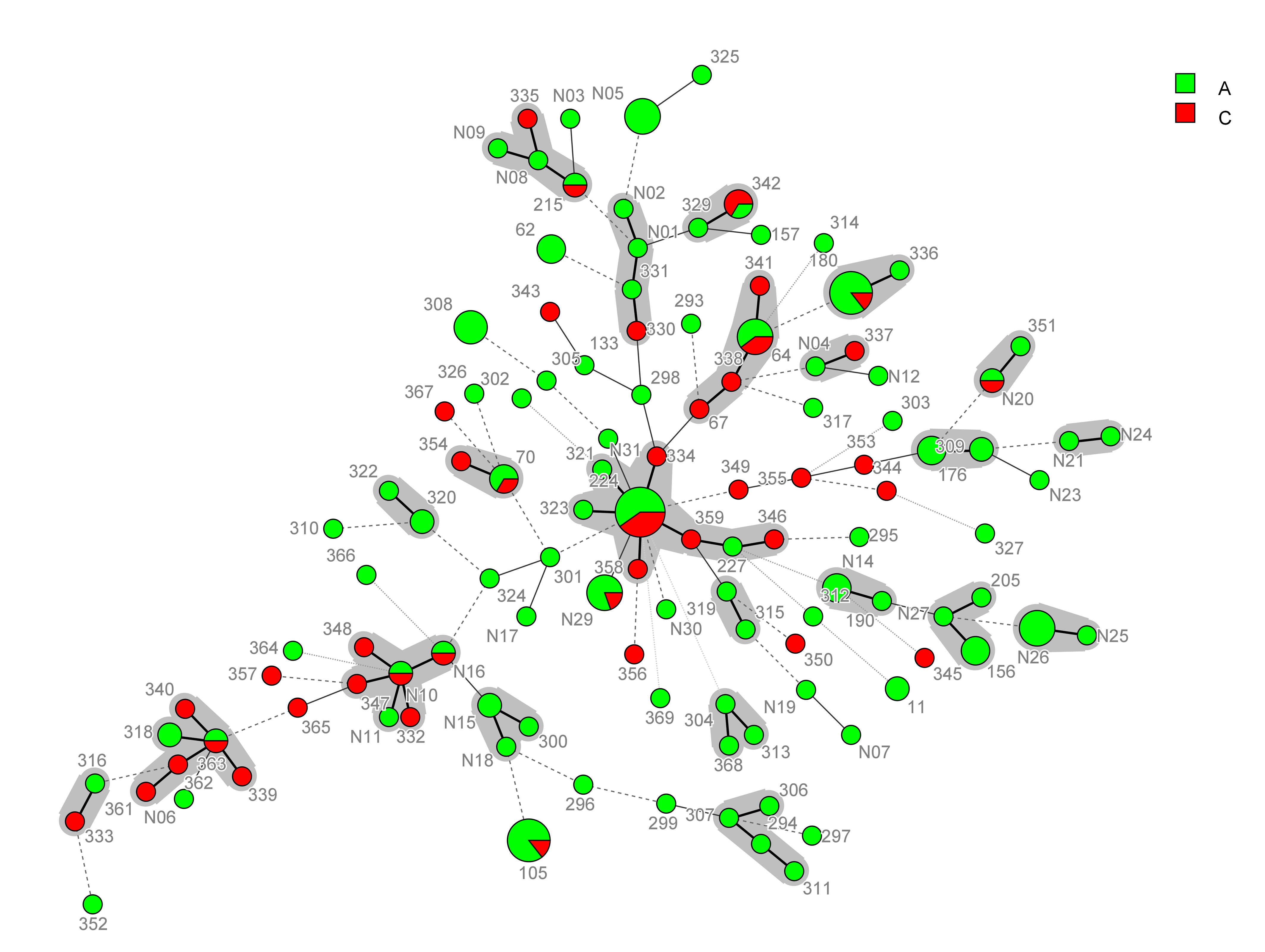

Supplement: FIGURE S2 — Population snapshot of M. catarrhalis based on allelic profiles of MLST in adults and children. Sequence types (STs) that shared alleles at ≥5 of the eight MLST loci were obtained from the M. catarrhalis MLST website, and a diagram was constructed by using Bionumerics. Each circle corresponds to a MLST ST, and different circle colors represent different population (A: Adult/C: children). The lines between circles indicate the similarity between profiles: bold line, seven of eight MLST alleles/MLVA loci in common; normal line, six alleles/loci in common; dashed line, five alleles/loci in common; dotted line, ≤4 alleles/loci. The gray halo surrounding the STs denotes STs belonging to different MLST clusters. Cluster 1: ST363/ST340/ST339/ST362/ST361/ST318, Cluster 2: STN10/STN11/STN16/ST332/ST348/ST347, Cluster 3: STN08/ST335/ST215/STN09, Cluster 4: ST224/ST227/ST323/ST334/ST346/ST358/ ST359/STN31, Cluster 5: STN25/STN26, Cluster 6: ST330/ST331/STN01/STN02, Cluster 7: ST64/ST67/ST338/ST341, Cluster 8: ST329/ST342, Cluster 9: ST180/ST336, Cluster 10: ST337/STN04, Cluster 11: STN20/ST351, Cluster 12: STN21/STN24, Cluster 13: ST176/ ST309, Cluster 14: ST156/ST205/STN27, Cluster 15: ST190/STN14, Cluster 16: ST315/ST319, Cluster 17: ST304/ST313/ST368, Cluster 18: ST294/ST306/ST307/ST311, Cluster 19: ST337/STN04, Cluster 20: ST300/STN15/STN18, Cluster 21: ST320/ST322, Cluster 22: ST70/ST354. [file Image_2.TIF]

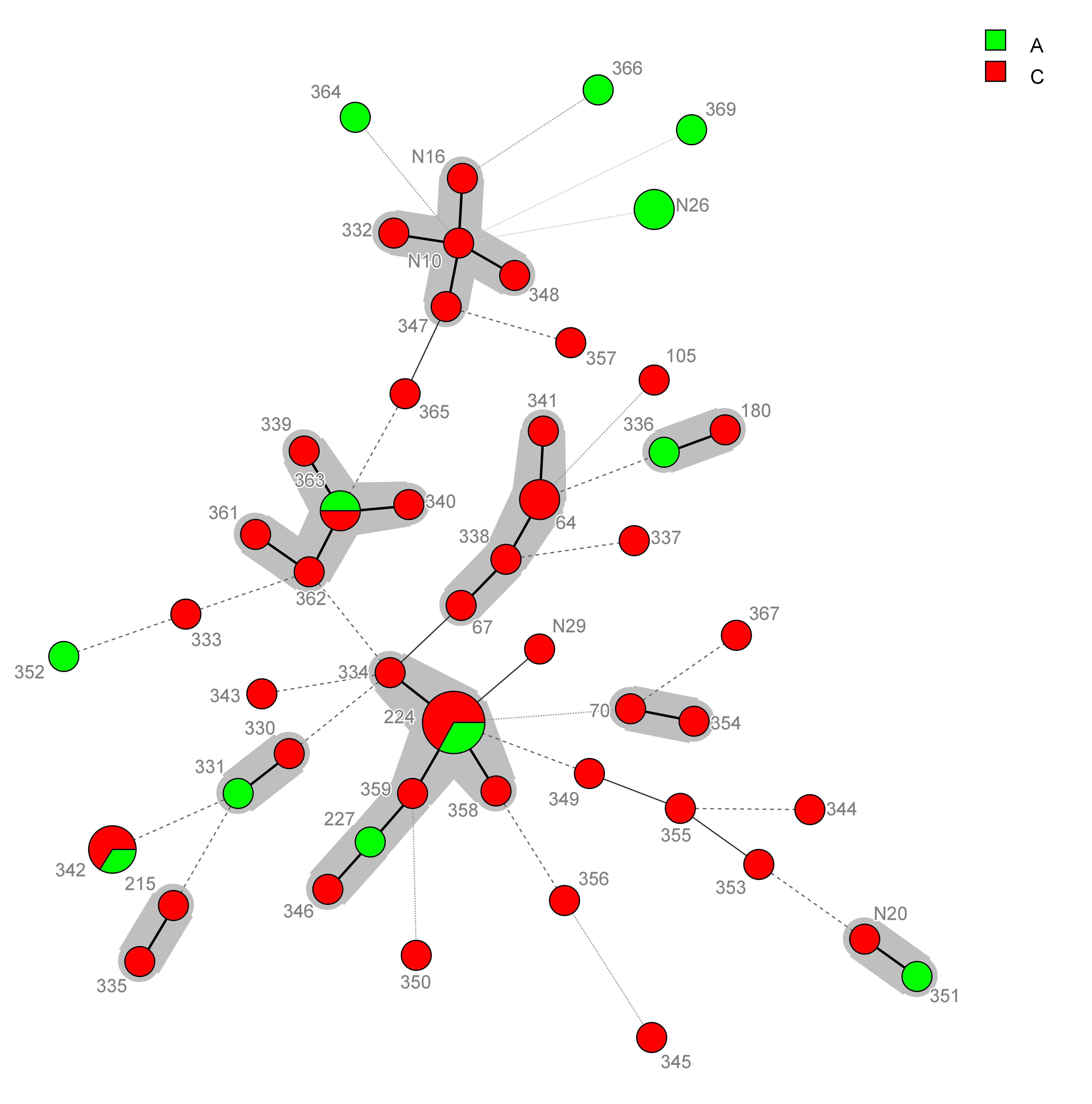

Supplement: FIGURE S3 — Population snapshot of M. catarrhalis based on allelic profiles of MLST in adults and children of FAHXU. Sequence types (STs) that shared alleles at ≥5 of the eight MLST loci were obtained from the M. catarrhalis MLST website, and a diagram was constructed by using Bionumerics. Each circle corresponds to a MLST ST, and different circle colors represent different population (A: Adult/C: children). The lines between circles indicate the similarity between profiles: bold line, seven of eight MLST alleles/MLVA loci in common; normal line, six alleles/loci in common; dashed line, five alleles/loci in common; dotted line, ≤4 alleles/loci. The gray halo surrounding the STs denotes STs belonging to different MLST clusters. [file Image_3.TIF]
